# Generation of a single-cycle pulse using a two-stage compressor and its temporal characterization using a tunnelling ionization method

**DOI:** 10.1038/s41598-018-38220-z

**Published:** 2019-02-07

**Authors:** Sung In Hwang, Seung Beom Park, Jehoi Mun, Wosik Cho, Chang Hee Nam, Kyung Taec Kim

**Affiliations:** 1 Center for Relativistic Laser Science, Institute for Basic Science, Gwangju, 61005 Korea; 2 Department of Physics and Photon Science, Gwangju Institute of Science and Technology, Gwangju, 61005 Korea

## Abstract

A single-cycle laser pulse was generated using a two-stage compressor and characterized using a pulse characterization technique based on tunnelling ionization. A 25-fs, 800-nm laser pulse was compressed to 5.5 fs using a gas-filled hollow-core fibre and a set of chirped mirrors. The laser pulse was further compressed, down to the single-cycle limit by propagation through multiple fused-silica plates and another set of chirped mirrors. The two-stage compressor mitigates the development of higher-order dispersion during spectral broadening. Thus, a single-cycle pulse was generated by compensating the second-order dispersion using chirped mirrors. The duration of the single-cycle pulse was 2.5 fs, while its transform-limited duration was 2.2 fs. A continuum extreme ultraviolet spectrum was obtained through high-harmonic generation without applying any temporal gating technique. The continuum spectrum was shown to have a strong dependence on the carrier-envelope phase of the laser pulse, confirming the generation of a single-cycle pulse.

## Introduction

Ultrashort laser pulses have become essential tools for studying ultrafast light-matter interactions^1–5^. The duration of the laser pulse is a key parameter for controlling strong-field processes, such as above-threshold ionization (ATI)^6^, high-harmonic generation (HHG)^7^, and frustrated tunnelling ionization (FTI)^8^. Tunnelling ionization, occurring every half optical cycle of the laser pulse, is the first step of those processes. In order to control the number of ionization events in such experiments, it is important to reduce the number of cycles in the pulse. The temporal confinement of the ionization allows a precise time-resolved measurement of light-matter interactions^9^. It also enables the generation of an isolated attosecond pulse through HHG^10^. Consequently, an efficient method for the generation and characterization of a single-cycle laser pulse is needed for the study and application of ultrafast optical science.

Tremendous efforts have been made to reduce the duration of a laser pulse down to a few cycles or to the single-cycle regime^1,11–14^. Obtaining such an ultrashort laser pulse requires spectral broadening and phase control over the spectrum. Hollow-core fibres have been widely used for spectral broadening of milli-joule laser pulses^15–21^. The spectrum of the laser pulse is broadened due to nonlinear interactions, such as the optical Kerr effect or ionization in a gas-filled hollow-core fibre. The output pulse from these fibres can have a spectrum broad enough to generate a single-cycle pulse; however, complicated phase structure develops during propagation in the nonlinear optical media. The laser pulse can be compressed down to the limit of only a few cycles when the second-order dispersion is compensated using chirped mirrors^15,16^. In order to further compress the pulse, third- and higher-order dispersion must be compensated using a higher-order chirped mirror^22,23^, material dispersion^24,25^, or pulse synthesizer^11,12^.

Spectral broadening of a laser pulse can also be achieved in a solid material. A solid material is vulnerable to optical damage due to self-focusing of a laser pulse^26–30^. Kung *et al*. avoided this self-focusing problem by using multiple thin glass plates. The multiple glass plates were arranged near the focus so that the self-focusing of the laser beam was balanced by the divergence of the laser beam. In this way, it was possible to compress the laser pulse down to the few-cycle limit when the second-order dispersion is compensated.

In this report, we present a two-stage compressor developed for the generation of a single-cycle laser pulse. The two-stage compressor consists of a hollow-core fibre and multiple thin glass plates; the spectrum of a laser pulse is broadened due to the Kerr effect in both cases, which universally occurs for all wavelengths. The arrangement of the two-stage compressor offers sufficient spectral broadening for the generation of a single-cycle laser pulse. It is more efficient than a two-stage compressor that uses two hollow-core fibres^31^, because the second stage of compression using multiple plates is insensitive to the alignment of the laser beam. Our measurements show that the development of higher-order dispersion is suppressed in the two-stage compressor, and single-cycle laser pulses can be generated by compensating the second-order phase with chirped mirrors. The temporal characterization of the single-cycle laser pulse was performed using the tunnelling ionization with perturbation for the time-domain observation of an electric field (TIPTOE) method^32^.

## Results

### Limitation of single-stage compression

**Figure 1:**
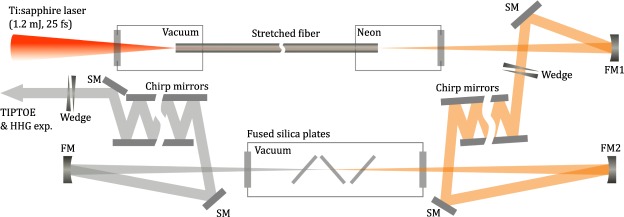
Experimental setup for single-cycle pulse generation and characterization. A laser pulse with an energy of 1.2 mJ was focused into a stretched hollow-core fibre. A focusing mirror (FM1) was used to collimate the beam after the fibre. Flat silver mirrors (SM) were used to steer the beam. A glass wedge was used to control the dispersion after the collimation of the beam, and an array of chirped mirrors (CM) was used to compensate for second-order dispersion after the wedge. The resulting 120-μJ pulse was focused with a focusing mirror (FM2) onto multiple thin fused-silica plates in a vacuum chamber; three fused-silica plates (50, 50, and 100 μm) were placed at Brewster’s angle in the vacuum chamber. Next, a second set of chirped mirrors as well as another glass wedge were used to control the dispersion of the pulses after the vacuum chamber. Finally, the compressed laser pulses were sent to the TIPTOE^32^ setup for temporal characterization or HHG experiments.

**Figure 2:**
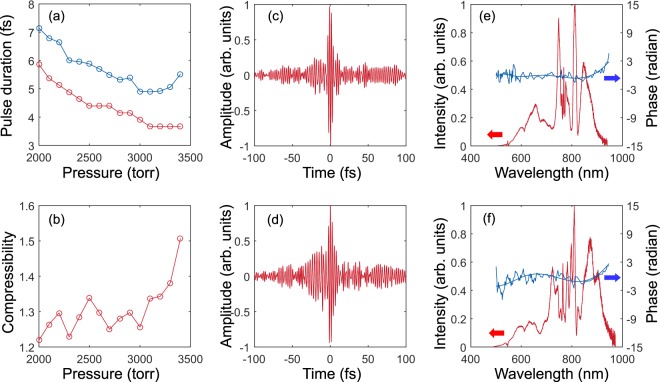
Pulse duration and spectra obtained at different Ne pressures in a hollow-core fibre. (**a**) The pulse duration, measured by the TIPTOE method^31^ after compensating for the second-order dispersion with the set of chirped mirrors, is indicated by the blue line, and the red line represents the transform-limited pulse duration estimated from an experimental spectrum. (**b**) The compressibility, calculated by dividing the measured pulse duration by the transform-limited pulse duration, on Ne pressure. (**c**,**d**) Electric field temporal amplitudes measured for Ne pressures of (**c**) 3000 and (**d**) 3400 torr. (**e**,**f**) Spectral intensities (red lines) and phases (blue lines) for Ne pressures of (**e**) 3000 and (**f**) 3400 torr.

The experimental setup for the two-stage compression is shown in Fig. 1. In order to determine the optimal pressure of the hollow-core fibre to minimize the pulse duration, the temporal profiles of the compressed laser pulse were measured using the TIPTOE method at various gas pressures. Once the pressure in the hollow fibre was set to a certain value, the temporal profiles were measured while adjusting the thickness of the glass wedge to find the shortest pulse duration. The shortest pulse duration (blue line) for different pressures are shown alongside their transform-limited pulse duration (red line) in Fig. 2(a). Since the spectral broadening increased with Ne pressure, the transform-limited pulse duration *τ*_TL_ gradually decreases with pressure up to 3400 torr. The actual pulse duration *τ*, however, decreases with pressure only up to 3000 torr, and the duration increases at pressures above 3000 torr. The compressibility of the fibre is defined as the ratio of the two durations (*τ/τ*_TL_), and its dependence on Ne pressure is shown in Fig. 2(b). Since the second-order phase of the laser pulse can be compensated using a set of chirped mirrors and a glass wedge, the increase of the compressibility indicates that a significant amount of higher-order dispersion of the pulse exists at high pressure.

The amount of the spectral broadening increases with the Ne pressure in the fibre, but there is a limit to the resulting reduction in the pulse duration. The temporal profiles of the laser pulses obtained at 3000 torr and 3400 torr are compared in Fig. 2(c,d). While the spectral phase is almost flat at 3000 torr (Fig. 2(e)), the spectral phase obtained at 3400 torr shows that the third-order phase developed during the nonlinear propagation in the fibre (Fig. 2(f)). In the time domain, the residual higher-order dispersion caused the formation of satellite pulses near the main pulse, as shown in Fig. 2(d). Thus, if the higher-order dispersion is not suppressed, the shortest duration that can be achieved using a single-stage compressor is limited.

### Theoretical calculation for a second pulse compressor

**Figure 3:**
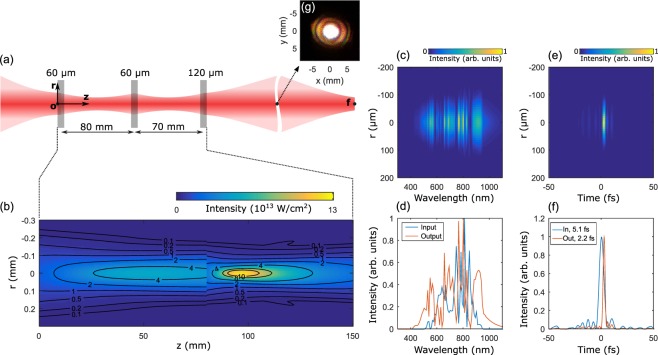
Nonlinear propagation through multiple glass plates. The nonlinear pulse propagation is solved numerically^33^. (**a**) Cylindrical symmetry along the propagation axis (*z*) is assumed in the calculation. The thicknesses of the glass plates are 60, 60, and 120 μm and their separations are 80 and 70 mm. (**b**) Intensity distribution of the laser beam from the first to the last glass plate; the numbers on the contour lines represent the intensity in units of 10^13^ W/cm^2^. (**c**) Spectra of the laser beam at the point ‘f’ as a function of ***r***. (**d**) Spectra of the laser beam at the point ‘o’ (input) and ‘f’ (output). (**e**) Temporal intensity profiles of the laser pulse at the point ‘f’ as a function of ***r***. (**f**) Temporal intensity profiles of the laser pulse at the point ‘o’ (input) and ‘f’ (output). (**g**) Beam profile measured at the far field after the second compressor.

The propagation of the laser beam through the multiple fused-silica plates in space and time was numerically solved near the multiple glass plates as illustrated in Fig. 3(a). The peak intensity of the laser beam is 2 × 10^13^ W/cm^2^ just before the first glass plate. Without the glass plates, the laser beam should have been focused at *z* = 8 cm. However, the laser beam was focused at *z* = 5 cm due to the self-focusing effect as shown in Fig. 3(b). The laser beam diverges after the first focus (*z* = 5 cm); it is focused again at the second focus (*z* = 9.6 cm), after propagation through the second glass plate, and it diverges again. After the propagation through the third plate, the laser beam diverges up to the far field. Then, it is assumed that the laser beam is refocused at the point ‘f’ in Fig. 3(a). The temporal profile and spectrum of the laser pulse are calculated at the point ‘f’. The spectrum is broadened (Fig. 3(c,d)). The duration of the laser pulse was estimated after compensating the second-order phase of the pulse as 2.22 fs (Fig. 3(e,f)), while the transform-limited duration is 2.19 fs. This numerical calculation shows that the higher-order phase is not much developed during the propagation through the multiple glass plates. In addition, the laser beam is very little distorted during the nonlinear propagation in the second compressor as shown in Fig. 3(c,e). Thus, a single-cycle pulse can be efficiently generated using our two-stage compressor.

### Generation of a single-cycle pulse using a two-stage compressor

A two-stage compressor was implemented experimentally. In order to compress the few-cycle laser pulse obtained from the hollow-core fibre to the single-cycle limit, multiple fused-silica plates were employed at the second stage of the compressor, as shown in Fig. 1. The input energy before the multiple glass plates was 120 μJ while the output energy was 90 μJ, which indicates a transmittance of 75%. The beam shape measured at the far field after the second compressor is shown in Fig. 3(g). According to the theoretical calculations shown in Fig. 3, we expect the duration to be near to that of a single cycle. Since conventional temporal characterization techniques rely on second-harmonic generation, it is difficult to measure the single-cycle pulse duration due to the limited bandwidth of a nonlinear crystal. Here, we employ a new temporal characterization technique called TIPTOE, in which a sub-cycle ionization event is used as a fast temporal gate to sample an additional weak field^32^. Since the technique can be applied over a broad wavelength range, it is an ideal tool for the temporal characterization of ultrashort laser pulses.

**Figure 4:**
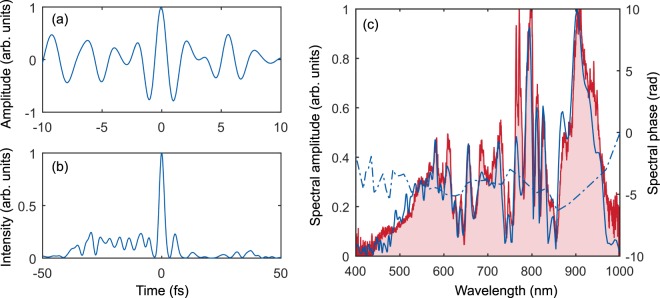
Temporal characterization of the single-cycle pulse obtained via a two-stage compressor. The temporal profiles of compressed pulses were measured using the TIPTOE method^32^. (**a**) Electric field amplitude and (**b**) intensity profiles; the measured duration of the pulse is 2.5 fs. (**c**) Spectral amplitude (blue line) and phase (blue dash-dotted line) obtained by taking the Fourier transform of (**a**); the experimentally-measured spectrum is indicated by the red line in part (**c**).

In the TIPTOE method, the laser pulse is divided into two pulses with different intensities to form a strong pulse that tunnel-ionizes air molecules and a weak pulse superposed with the strong pulse. The modulation of the ionization yield, measured as a function of the time delay between the two pulses, represents the temporal profile of the electric field of the weak pulse. The temporal profile of the electric field, obtained from the modulation of the ionization yield, is shown in Fig. 4(a). The central wavelength of the main pulse is 650 nm (for which wavelength, 1 optical cycle is 2.17 fs). The duration of the main pulse is 2.5 fs as shown in Fig. 4(b) which corresponds to 1.15 optical cycle at 650 nm. The main pulse contains 51% energy. Although the growth of pre-pulses is not observed in the theoretical calculations shown in Fig. 3, the pre-pulses become more prominent after the second compressor in the experiment. The existence of the pre-pulse would be a serious problem for some applications; however, applications like HHG would not be much affected by such structure since the amount of ionization caused by the pre-pulses is not significant when compared to that of the main pulse. The spectral amplitude and phase of the laser pulse after the second compressor (blue lines in Fig. 4(c)) were obtained by taking the Fourier transform of the temporal laser field. The spectrum retrieved in this way agrees well with the experimentally measured spectrum (red line), supporting the validity of the TIPTOE measurement.

### High harmonic generation with a single-cycle laser pulse

**Figure 5:**
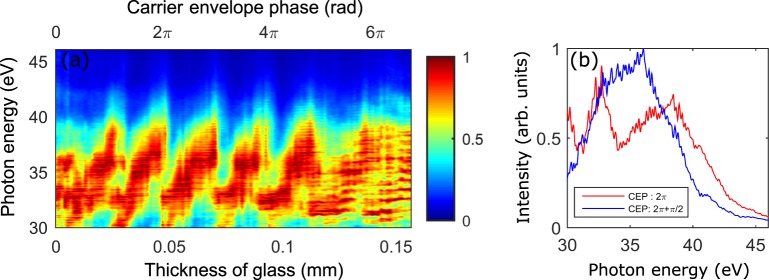
High-harmonic spectra generated in Ar using single-cycle laser pulses. High-harmonic spectra were generated while adjusting the insertion thickness of the glass wedges to control carrier-envelope phase (CEP). (**a**) CEP change with respect to the insertion thickness is shown above the graph. A single-cycle pulse with 45 μJ of energy was focused (*f* = 10 cm) onto the Ar-gas target. The peak intensity of the laser beam at the target is estimated to be 1.6 × 10^14^ W/cm^2^. (**b**) Comparison of high-harmonic spectra generated with CEPs of 2π (red line) and 2.5π (blue line).

An intense single-cycle laser pulse can confine an ionization event within a half cycle at the peak of the laser pulse, which can be used for the generation of an isolated attosecond pulse^11^. To see the effect of a single ionization event, we performed a HHG experiment using the single-cycle pulses obtained from the two-stage compressor. The central part of the laser beam with 45 μJ of energy was focused onto an argon gas target using a focusing mirror with a 10-cm focal length. We controlled the dispersion and carrier-envelope phase (CEP) of the single-cycle pulses using a pair of glass wedges. High-harmonic spectra were recorded for different thicknesses of the glass wedge. The high harmonic spectra are continuous, as shown in Fig. 5. The amplitude values of the harmonic spectra were greatly modulated as the CEP of the laser pulse changed, a typical feature of harmonic spectra obtained with single-cycle laser pulses. These measurements confirm the generation of single-cycle pulses.

## Discussion

We demonstrated a method for generating and characterizing single-cycle laser pulses. Single-cycle laser pulses were obtained using a two-stage compressor consisting of a hollow-core fibre and subsequent multiple fused-silica plates. Temporal characterization was achieved by applying the TIPTOE method. It was found that the two-stage compressor mitigated the development of higher-order dispersion, making compensation of higher-order dispersion unnecessary. The initial laser pulse duration of 25 fs was compressed down to 2.5 fs, as expected from numerical calculations. The continuum harmonic spectrum exhibited strong dependence on the CEP of laser pulses, supporting our characterization of the single-cycle laser pulse via the TIPTOE method. The ultrashort laser pulse will be an important tool for studying time-resolved phenomena. In addition, it will be used as a versatile tool for controlling the strong-field processes such as ATI, HHG, and FTI.

## Methods

A carrier-envelope-phase-stabilized 25-fs pulse at 800 nm from a titanium sapphire laser (Femtolasers, Femtopower X CEP) was used to perform all experiments. The laser beam was focused (f-number = 222) into the first compressor which is consists of a stretched hollow-core fibre (500-μm inner diameter), chirped mirrors (Ultrafast innovation, HD PC37_4) and glass wedges (Femtolasers, BK7, 8°) as shown in Fig. 1. The entrance side of the fibre was connected to a vacuum pump, while the other side was filled with Ne gas to maintain a pressure gradient in the fibre. The pressure of the fibre was stabilized by the pressure controller (Alicat, PC-100PSI-D). The input energy of the hollow-core fibre was 1.2 mJ while the output energy was 0.8 mJ after the fiber, showing a transmittance of 67%. Due to the long distance between the first and the second compressor (~10 m) and the thick glass wedge, 32 chirped mirrors (16 pairs) are used. The energy of the laser beam was measured as 320 μJ after the chirped mirror and glass wedges. The iris was placed at the entrance of second stage compressor to get the optimal peak intensity of laser. The laser from the first compressor was focused using a 1.5 m focal length mirror into the second compressor. Three fused silica plates (50, 50, and 100 µm) were placed at Brewster’s angle in a vacuum chamber. The output energy was measured as 90 μJ, showing the transmittance of 75%. Most of loss was caused by the glass windows of the vacuum chamber which was placed perpendicularly with respect to the incident light direction. 16 chirped mirrors (Ultrafast innovation, HD PC37_4) are used after the second compressor. The spectra of the laser pulses shown in Figs 2 and 4 were measured by a grating-based spectrometer (Ocean optics, USB4000). The CEP of the single-cycle pulse was controlled by a glass wedge (Femtolasers, Fused silica, 2°48′) for HHG experiment. High harmonic spectra were measured using a chevron MCP imaging detector (Photonis) and a CMOS camera (PCO, Edge).

In order to study the propagation of a laser pulse in a second pulse compressor, the nonlinear propagation of the laser pulse through multiple fused-silica plates and vacuum spaces was analysed by solving the nonlinear pulse propagation equation in three dimensions (using Eq. 119 in ref.^33^). In the calculation, a nonlinear index *n*_2_ of 3 × 10^−16^ cm^2^/W was used for the description of the Kerr effect in the medium. The nonlinear absorption coefficient for the multiphoton absorption was adjusted to give the absorption value (5%) observed in the experiment. Experimental parameters, including pulse intensity, beam size at the focus (200 μm), and the separation of the glass plates (8 and 7 cm), are used in the calculation. Since cylindrical symmetry along the propagation axis (*z* axis in Fig. 3) is assumed, the glass plates were orthogonal to the propagation direction in the calculation model, while they are placed at Brewster’s angle in the experiment; therefore, the thickness of the glass was increased by 20% for the three plates (60, 60, and 120 μm) in the calculation to compensate for the angle difference. Since all parameters for the calculation were chosen to be as close as possible to those of the experimental conditions, the propagation of the laser pulse in the multiple glass plates could be accurately modelled.
